# Analytical Problems with Preparation of Paraspinal Tissues from Patients with Spinal Fusion for Analysis of Titanium

**DOI:** 10.3390/molecules26082120

**Published:** 2021-04-07

**Authors:** Jan Sawicki, Anna Danielewicz, Magdalena Wójciak, Michał Latalski, Agnieszka Skalska-Kamińska, Katarzyna Tyszczuk-Rotko, Ireneusz Sowa

**Affiliations:** 1 Department of Analytical Chemistry, Medical University of Lublin, 20-093 Lublin, Poland; jansawicki@umlub.pl (J.S.); magdalenawojciak@umlub.pl (M.W.); agnieszka.skalska-kaminska@umlub.pl (A.S.-K.); 2 Department of Paediatric Orthopaedics, Medical University of Lublin, 20-093 Lublin, Poland; anna.danielewicz@umlub.pl (A.D.); michallatalski@umlub.pl (M.L.); 3 Faculty of Chemistry, Institute of Chemical Sciences, Maria Curie-Skłodowska University in Lublin, 20-031 Lublin, Poland; ktyszczuk@poczta.umcs.lublin.pl

**Keywords:** titanium implants, optimization of digestion, paraspinal tissue, microwave mineralization, carbon heating block

## Abstract

Preparation of paraspinal tissue of patients with implants for elemental analysis is a challenge because it contains titanium in the ionic form, as well as metallic debris. Most literature reports focus on dissolving the tissue, but the impact of digestion conditions on metallic debris of Ti has not been investigated. In our work, various digestion conditions, including systems, compositions of oxidising mixture, and time, were tested aiming (i) to digest the tissue without digestion of metallic titanium to quantify soluble Ti and (ii) to digest metallic titanium debris to asses total Ti content in tissue. The experiments were performed in a closed mode using a microwave-assisted system and a carbon heating block. Our study revealed that total digestion of titanium was impossible in the tested conditions and the maximal level of digested titanium was below 70%. The mineralisation with the use of concentrated nitric acid was optimal to prepare paraspinal samples to analyse the soluble titanium form because metallic titanium passivated and did not migrate to the solution. The elaborated conditions were applied to determine titanium ion in the periimplant tissue of patients with three different titanium-based surgical systems, including traditional growing rod (TGR), guided growth systems (GGS), and vertical expandable prosthesis titanium rib (VEPTR).

## 1. Introduction

Surgical treatment of scoliosis involves spinal fusion mostly with the use of titanium-based biomaterials. Titanium is a relatively inert metal; however, a long-term exposure of implants to the biological environment leads to progressive wear and unfavourable processes, e.g., corrosion and mechanical damage caused by friction and abrasion. Consequently, titanium is gradually released into surrounding tissues and metal particles may cause local allergy and inflammation. Moreover, soluble titanium may migrate to blood and distant organs [1]. Therefore, analysis of the titanium level in various tissues of patients with implants is a relevant research issue and has great significance from the point of view of human health [1,2,3,4].

The general requirement of modern techniques for elemental analysis, such as atomic absorption spectrophotometry and inductively coupled plasma spectrometry, is the necessity to convert a solid sample into a representative solution. This laborious and time-consuming step depends on the type of sample, the complexity of matrix, and the nature of the analyte. There are many reports on determination of titanium in patients subjected to treatment with implants; however, they are mostly focused on blood and tissues distant from the surgical place [1]. However, paraspinal tissue adjacent to implants is specific, as it contains titanium in a soluble ionic form, as well as metallic debris. To assess both forms, the conditions of sample preparation should be carefully chosen. Firstly, the organic matrix should be fully decomposed without digestion of metallic debris to quantify the content of titanium dissolved in the tissue. Secondly, metallic solids should be dissolved to estimate the total titanium in tissue.

In literature, different strategies, including various oxidising mixtures and types of digestion, were described for preparation of tissue containing both forms of Ti for elemental analysis; however, no justification for the choice of the method was included [3,4,5,6,7,8,9,10,11,12]. Moreover, there are no systematic studies on the influence of the conditions used on particular Ti forms. Therefore, the aim of our work was to test experimentally the effect of different compositions of the oxidising mixture, including various concentrations of nitric acid and its mixtures with perchloric acid and hydrogen peroxide, as well as the temperature and time of the mineralization process on the effectiveness of titanium and tissue digestion. The digestion was performed in a closed mode using a microwave-assisted system and a carbon heating block.

## 2. Results

Before elemental analysis, the organic matrix should be decomposed by oxidation to gaseous products because non-decomposed tissue would disturb nebulisation of the solution, and the analyte would not be fully released from the tissue. Wet digestion with nitric acid and its combination with hydrogen peroxide, sulphuric acid, or perchloric acid is the most common dissolution method; however, the concentration of reagents, as well as the type and temperature of the process, are crucial factors influencing the effectiveness.

In our investigation, mineralisation in a closed system was chosen. When the process is conducted at atmospheric pressure in open systems, the boiling temperature of the mixture composed of nitric acid and water is insufficient to oxidise the organic matrix completely. The other parameters of digestion were experimentally tested. Two aspects were taken into consideration to find the proper conditions: (i) digestion of tissue without digestion of metallic titanium and (ii) digestion of metallic titanium debris. The results were expressed as a percentage of Ti found in the solution relative to Ti added to the tissue (% recovery). The minimal or maximal level of Ti recovery was regarded suitable to the aim of the study.

### 2.1. Digestion in the Carbon Heating Block

**Figure 1:**
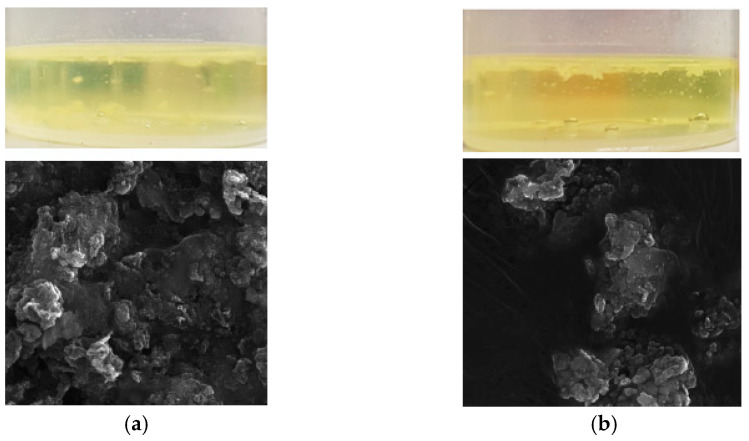
Visual and scanning electron microscopy images of the digestion solution after mineralisation: (**a**) non-decomposed tissue; (**b**) partially decomposed tissue.

Initially, the carbon heating block was chosen for our study. It is a relatively inexpensive and easily operated system. Its great advantage is the disposable vessels with screw-on filters, which minimise the risk of contamination. The vessels were closed during mineralisation (the volume of the liquid did not exceed 5 mL to prevent an excessive increase in pressure and damage to the vessels). Different combinations of the oxidising mixture, including nitric acid and mixtures of nitric acid with hydrogen peroxide and perchloric acid in various ratios, were tested. The totality of matrix digestion was assessed visually or with scanning electron microscopy if necessary. The examples of the solutions after digestion are shown in Figure 1.

In our research, different concentrations of nitric acid were tested. Nitric acid is commonly used for digestion of various tissues; however, the concentration of the acid should be carefully chosen because nitric oxides formed during digestion are harmful for the environment, and a strongly acidic solution can damage parts of the apparatus. In turn, an insufficient concentration of acid results in incomplete decomposition of the organic matrix.

**Figure 2:**
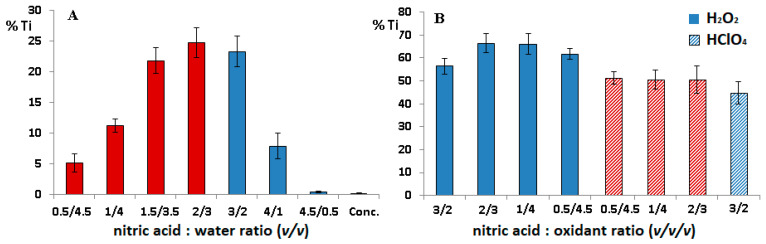
Recovery of titanium (%) using (**A**) different concentrations of nitric acid; (**B**) different mixtures composed of nitric acid/hydrogen peroxide and nitric acid/perchloric acid. Bars marked in red colour indicate that the matrix was not fully decomposed.

As can be seen in Figure 2A, the recovery of titanium at the lowest concentration of nitric acid was relatively high (ca. 5%); moreover, up to 7.5 mol/L of nitric acid, the matrix was not fully decomposed (bars in Figure 2 marked in red colour). However, the solutions obtained at the higher concentrations were transparent, and no fragments of tissue were visible under the microscope, and the recovery of titanium significantly decreased as a result of passivation of Ti particles. Thus, it can be concluded that digestion of tissue with Ti debris should be carried out with the use of concentrated nitric acid to assess the soluble titanium form.

On the other hand, the maximal level of digested titanium did not exceed 25%; thus, to increase the oxidative power of the mixture, the effect of addition of perchloric acid and hydrogen peroxide on titanium digestion was assessed. Both reagents increased the recovery of Ti significantly; however, the value did not exceed 70%, and the value was insufficient to determine total Ti content in the tissue. The examples of the results are presented in Figure 2B. Moreover, the prolongation of the mineralisation time even up to 240 min did not enhance the digestion of titanium; therefore, the system is not suitable for preparation of tissues with metallic debris for analysis of Ti.

### 2.2. Microwave Assisted Mineralisation

In our further investigation, a microwave-assisted system was used to prepare titanium-containing samples for the elemental analysis. Although the cost of such equipment is relatively high, it is very effective and is recommended for hard-to-digest materials.

**Figure 3:**
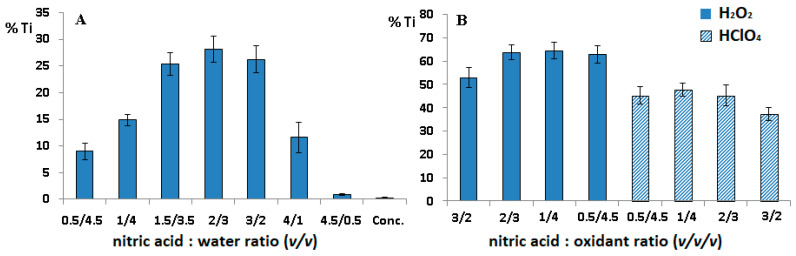
Recovery of titanium (%) using (**A**) different concentrations of nitric acid; (**B**) different mixtures composed of nitric acid/hydrogen peroxide and nitric acid/perchloric acid.

As in the procedure described in Section 2.1, the impact of different concentrations of nitric acid and its mixtures with hydrogen peroxide and perchloric acid on the digestion of titanium debris and muscle tissue was assessed. The results are shown in Figure 3.

As can be seen, the percentage of digested titanium was slightly higher than that for the heating block; the minimal value was ca. 0.2%, and the maximal one did not exceed 70%. This means that the soluble form of Ti can be determined when concentrated acid is used, and the conditions are not suitable for estimation of metallic titanium. However, it should be noted that the microwave-assisted process was more effective in tissue digestion even at a low acid concentration, which is beneficial from the point of view of consumption of Suprapur^®^ reagents.

Since the oxidation potential of nitric acid strongly depends on temperature, the time-temperature working program of the apparatus was further modified. Different variants were tested, including higher/lower temperature and increased time; however, no improvement of the results were noted.

It should also be mentioned that a serious disadvantage of the microwave system is the necessity to washout and digest the Teflon vessel after each use, which prolongs the experimental procedure. Moreover, residues of the matrix or analyte sometimes penetrate into the vessel walls, resulting in memory effects, and an excessive concentration of nitric acid decreases considerably the lifetime of polymer vessels.

### 2.3. Digestion with the ISO Method

The samples were also digested in both systems using the ISO method dedicated to Ti. The method is based on mineralisation with sulphuric acid with addition of ammonium sulphate. However, the recovery of titanium did not exceed 60%. Moreover, the organic matrix was not fully decomposed in the heating block even if the digestion was repeated, as suggested in the procedure.

### 2.4. Analysis of Titanium Ions in Paraspinal Tissue of Patients with Titanium Implants

**Figure 4:**
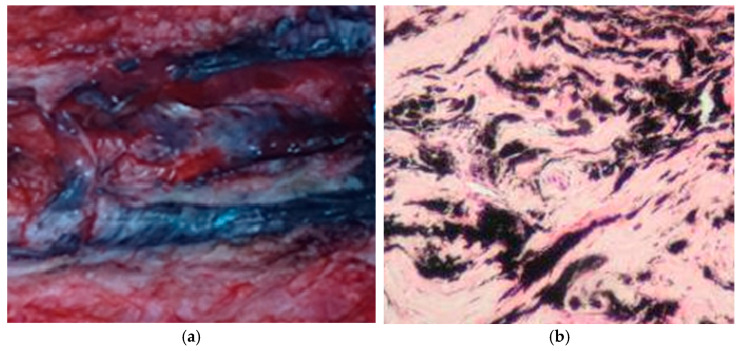
(**a**) Sample of paraspinal tissue from patients with implants; (**b**) histopathological preparation showing black metal deposits in the intercellular spaces of tissue.

The samples of tissue of patients with different titanium-based surgical systems to correct spinal deformity (traditional growing rod (TGR), guided growth systems (GGS), and vertical expandable prosthesis titanium rib (VEPTR)) were provided by the Paediatric Orthopaedic Clinic of the Medical University of Lublin. Metallosis was clearly visible in all samples. The example is shown in Figure 4.

Homogenised samples were digested using concentrated nitric acid in the carbon heating block and microwave systems to estimate the soluble form of titanium. All mineralisates were clear without opalescence, which indicates that the organic matrix was fully decomposed, as confirmed by the microscopic observation. The results of Ti quantification are shown in Table 1. As can be seen, the differences between the results obtained for samples prepared using both systems were not statistically significant.

## 3. Discussion

There are many methods for preparation of tissues for elemental analysis, and dissolution of the tissue is usually not a big challenge. Treatment with acids or bases is the most common procedure. It is effective if appropriate concentrations of reagents and methods of digestion are chosen [3]. However, proper preparation of tissues containing titanium as metallic particles and soluble ions is very difficult. Two approaches can be useful to resolve this problem: one of them includes full solubilisation of the sample with metallic debris, and the other one includes digestion of tissue without dissolution of Ti particles, filtration, and quantification of only the soluble form of the analyte [3,4,5]. Ti is a chemically inert metal and, as shown in our study, the conversion of total Ti into the soluble ion form using standard digestion methods, such as heating and microwave mineralization, is impossible. Thus, the second resolution seems to be better. The literature review has revealed that treatment with nitric acid at different concentrations is the most common procedure for periimplant tissue; however, most reports focused on dissolving the tissue and the impact of digestion conditions on metallic Ti debris was not investigated. Our investigation proved that Ti is partially soluble, even in a mild acidic solution, when mineralisation is supported by microwaves or heating. On the other hand, heating with the use of a low concentration of acid did not decompose the tissue effectively. Concentrated nitric acid, which prevents the digestion of Ti by covering the particle surface with titanium oxide, is the best solvent for preparation of tissue to analyse only the ionic titanium form.

Treatment with strong bases is an alternative to acidic digestion; this approach was used by Kosssovsky et al. [13] and Shanbhag et al. [14] to decompose tissue for quantification of metallic particles; however, the high density of the solution obtained causes problems with nebulisation and reaction with quartz elements; therefore, it is not recommended for inductively coupled plasma spectrometry determination.

On the other hand, enzymatic treatment is sometimes used for digestion [15,16], but Campbell et al. [17] have found that it is not fully effective to decompose the organic matrix. Table 2 summarises the experimental conditions applied for preparation of titanium-containing tissues for elemental analysis.

## 4. Materials and Methods

### 4.1. Chemicals

Sixty-five percent nitric acid Suprapur^®^, 31% hydrogen peroxide Suprapur^®^, 60% perchloric acid EMSURE^®^, sulphuric acid, and ammonium sulphate were purchased from Merck (Darmstad, Germany). Deionised water (DI water) was obtained from the Direct-Q^®^ 3 UV water purification system (Merck).

### 4.2. Sample Preparation

For the sample, 216 ± 12 mg of pork shoulder and 5.15 ± 0.23 mg of metallic titanium powder were weighed into digestion vessels, and 5 mL of a relevant oxidation mixture was then added. Tissue without Ti was used as a control. A microwave-assisted pressure digestion system (TOPwave, Analytik Jena, Jena, Germany) and heating block (DigiPREP, SCP Science, Baie-D’Urfé, Quebec, Canada) were used for sample digestion. Microwave digestion was carried out using CX 100 TFM-PTFE vessels and program specified in Table 3.

Digestion in the heating block was performed for 2 h at a temperature of 120 °C using closed 50-mL DigiTUBEs (SCP Science, Baie-D’Urfé, QC, Canada). After digestion, the solution was filtered with a DigiFILTER (SCP Science) and a vacuum pump Rocker 300C into clean DigiTUBEs and filled up to 10 mL with DI water.

Final conditions used for preparation of paraspinal tissues: 5 mL of concentrated nitric acid was added to 200 mg of tissue, and two mineralisation variants were applied, including digestion in heating block (2 h at a temperature of 120 °C) and microwave digestion, according to program given in Table 3 (in step 2, temperature was 180 °C, and time was 5 min). 

### 4.3. Analysis of Titanium Ions

Transparent solutions were then diluted 1000 times with DI water and titanium concentrations were quantified using inductively coupled plasma optical emission spectrometry (ICP-OES) (PlasmaQuant PQ 9100 Elite, Analityk Jena, Jena, Germany). The ICP-OES working conditions are presented in Table 4. The titanium standard solution was prepared using 1000 mg Ti, (NH_4_)_2_TiF_6_ in water Titrisol^®^ (Merck).

## 5. Conclusions

In the present study, the conditions for preparation of paraspinal tissue from patients with titanium implants were experimentally chosen taking into account two aspects, including (i) digestion of metallic titanium present near the implants and (ii) decomposition of tissue without digestion of Ti, which allowed assessment of the total Ti and the soluble form of Ti, respectively. Our study revealed that microwave digestion was more effective in dissolution of the organic matrix at a lower concentration of nitric acid; however, microwave only slightly supported the digestion of metallic titanium. Moreover, total digestion of titanium was impossible, irrespective of the systems used and the composition of the oxidizing mixture; therefore, the quantification of metallic Ti failed. The microwave system, as well as the heating block, can be successfully used to prepare periimplant tissue for analysis of the soluble form of Ti when concentrated nitric acid is used, as it prevents the digestion of metallic debris as a result of passivation. 

## Figures and Tables

**Table 1:** Content of titanium ions (*n* = 3) ± SD in paraspinal tissue of patients with different chirurgical systems. The samples were digested using the heating block and microwave systems.

Chirurgical System	Heating Block	Microwave Systems
TGR	0.551 ± 0.0591 mg/g	0.598 ± 0.0610 mg/g
GGS	1.55 ± 0.123 mg/g	1.77 ± 0.122 mg/g
VEPTR	0.954 ± 0.111 mg/g	1.10 ± 0.102 mg/g

**Table 2:** Experimental conditions applied for preparation of titanium-containing tissues.

Material	Digestion Mixture	Digestion Method and Conditions	Ref.
samples from patients with a spine implant; beef, pork samples, and CRM of the liver spiked with Ti ions	concentrated HNO_3_	microwave assisted digestion 160 °C 5 min 180 °C 5 min 200 °C 5 min	[6]
tissue from patients with elbow arthroplasty	concentrated HNO_3_	180 °C	[4]
periprosthetic tissue	10 M NaOH	72 h at 70 °C with stirring at 350 rpm	[18]
tibia tissues of rats with metallic implants	concentrated HNO_3_	200 W 8 min	[7]
dried hip tissues	50% H_2_O_2_, drying and dissolving in 2 mL of HNO_3_	approximately 30 h	[19]
tissue from patients with a Ti-alloy hip prosthesis	4M KOH	56 °C 48 h (two times)	[14]
tissue from patients with a hip or knee implant	concentrated HNO_3_	48 h at room temperature	[3,5]
tissue of patients with femoral endosteolysis and hip arthroplasty	concentrated HNO_3_ and HCl (3:1)	140 ℃	[20]
tissue of patients after total hip arthroplasties	77% HNO_3_	no information	[8]
lymphoreticular tissues	HNO_3_ and HClO_4_	no information	[9]
spleen, gastronemious muscle, lung from rabbits with a titanium implant	no information	Microwave digestion	[21]
samples of pig jaws with titanium dental implants	HNO_3_ and HF (7:1)	microwave digestion (160 ℃ 10 min, 190 ℃ 20 min, 50 ℃ 10 min)	[10]
mucosa surrounding the mandibular endoprosthesis in *Macaca fascicularis*	65% HNO_3_, 95% H_2_SO_4_, 30% H_2_O_2_ (3.5:0.5:0.5 *v/v/v*)	200 °C 40 min	[22]
human jawbones	sub-boiled distilled HNO_3_	170 °C 12 h	[11]
suspension of submucosal plaque from implants with periimplantitis and healthy implants	concentrated HNO_3_: H_2_O (50:50 *v/v*) with trace amount of HF	power 800 W (100%) ramp 15 min to 100 °C, 45 min	[12]
tissues of rats with Ti6Al4V implants	no information	microwave digestion	[23]

**Table 3:** Microwave digestion parameters.

Step	Temperature [°C]	Pressure [bar]	Ramp [min]	Time [min]	Power [%]
1	160	50	5	5	90
2	160–200	50	1	2–10	90
3	50	0	1	10	0
4	50	0	1	10	0
5	50	0	1	1	0

**Table 4:** Inductively coupled plasma optical emission spectrometry (ICP-OES) working conditions.

Analysis Line	334.9410 nm
Read time	3 s
Plasma power	1300 W
Plasma gas	14.0 L/min
Auxiliary gas flow	0.5 L/min
Nebuliser gas flow	0.6 L/min
Plasma monitoring direction	axial
Evaluations pixels	3 pix
Calibration curve range	0–1000 µg/L

## Data Availability

The data presented in this study are available on request from the corresponding author.

## Sample Availability

Samples are available from the authors.
